# A synthetic glycosaminoglycan reduces sinonasal inflammation in a murine model of chronic rhinosinusitis

**DOI:** 10.1371/journal.pone.0204709

**Published:** 2018-09-25

**Authors:** Jeremiah A. Alt, Won Yong Lee, Brock M. Davis, Justin R. Savage, Thomas P. Kennedy, Glenn D. Prestwich, Abigail Pulsipher

**Affiliations:** 1 Division of Head and Neck Surgery, Rhinology–Sinus and Skull Base Surgery Program, Department of Surgery, University of Utah School of Medicine, Salt Lake City, Utah, United States of America; 2 GlycoMira Therapeutics, Inc., Salt Lake City, Utah, United States of America; 3 Pulmonary Diseases Critical Care and Environmental Medicine, Tulane University School of Medicine, New Orleans, Louisiana, United States of America; 4 Department of Medicinal Chemistry and Center for Therapeutic Biomaterials, University of Utah, Salt Lake City, Utah, United States of America; University of Pittsburgh, UNITED STATES

## Abstract

Chronic rhinosinusitis (CRS) is characterized by sustained mucosal inflammation, impaired mucociliary clearance, loss of cilia and epithelial barrier breakdown, and tissue remodeling. Certain glycosaminoglycans inhibit various inflammatory mediators, suppress bacterial growth, and provide important functions in mucosal tissue repair and mucociliary clearance. Herein, we evaluated the effects of a synthetic glycosaminoglycan, GM-1111, on the clinical signs and inflammatory tissue changes associated with CRS in mice. CRS was generated by repeated intranasal applications of *Aspergillus fumigatus* (*A*. *fumigatus*) extracts over 4 weeks. Mice were then intranasally administered GM-1111 (600 μg per dose, 5 times a week) or vehicle (phosphate buffered saline, PBS) for an additional 4 weeks while still being given *A*. *fumigatus* extracts to maintain a chronic inflammatory environment with acute exacerbations. Clinical signs indicative of sinonasal inflammation were recorded throughout the study. After 9 weeks, whole blood and sinonasal tissues were harvested for hematological, histological, and biochemical examination. The clinical signs, white blood cell counts, tissue markers of sinonasal inflammation, and histological changes caused by *A*. *fumigatus* extract administration were compared to the healthy (PBS vehicle) and GM-1111-treated groups (n = 12 per treatment group). Compared to vehicle-treated animals, animals treated with GM-1111 demonstrated significant reductions in clinical signs (*p*<0.05), degenerative tissue changes, goblet cell hyperplasia, inflammatory cell infiltration (*p*<0.01), innate immunity- (*tlr2*, *tlr4*, *myd88*, *il1b*, *tnfa*, *il6*, and *il12*) and adaptive immunity-associated (*ccl11*, *ccl24*, *ccl5*, *il4*, *il5*, and *il13*) cytokine gene expression (*p<*0.05 to *p*<0.0001) in sinonasal tissues, and serum IgE levels (*p*<0.01). Our data suggest that GM-1111 significantly reduces local and systemic effects of CRS-associated sinonasal inflammation.

## Introduction

Chronic rhinosinusitis (CRS) is a common and debilitating inflammatory condition affecting the nose and paranasal sinuses of up to 15% of the worldwide population.[1,2] The most complete and current evidence-based recommendations for the medical management of CRS include a combination of saline irrigation, topical intranasal corticosteroid sprays, and depending on the phenotype, antibiotics and oral steroids.[2,3] Despite having a myriad of medical treatment options, a large percentage of patients remain unresponsive and experience severe exacerbations that require surgical intervention,[4] underscoring the need for more effective therapeutics.[5,6]

The lack of effective medical therapies to treat CRS is due to its complex pathophysiology, as CRS is a multifactorial disease with many possible etiologies. Historically, CRS has been classified into two major phenotypes characterized by the presence (CRSwNP) or absence (CRSsNP) of nasal polyps. Increasing evidence, however, supports that multiple endotypes mediated by unique or mixed inflammatory pathways exist in CRSsNP and CRSwNP.[7–10] Eosinophil-dominated endotypes associated with an underlying mechanism of allergy-mediated hypersensitivity are particularly challenging forms of noninvasive CRS that commonly occur in recalcitrant patients with CRSwNP. These patients usually demonstrate increased blood and tissue eosinophils and CD4-positive T cells, as well as elevated serum levels of immunoglobulin E (IgE) and tissue levels of eosinophil-specific cytokines and chemokines.[1,9,11] Experts agree that the elevation of these inflammatory endpoints may be the result of maladaptive immune signaling, triggered by impaired mucociliary function and epithelial cell barrier breakdown.[12,13] The sinonasal epithelium is comprised of ciliated cells and mucus-secreting goblet cells that function as the primary defense against pathogens due to their ability to limit activation of the innate immune response via pattern recognition receptors such as the Toll-like receptors (TLRs).[8,14] Investigations have demonstrated that inappropriate regulation of TLR2 and TLR4 signaling contribute to the increased inflammation seen in CRS.[15–19] The TLRs are potent innate immune receptors involved in the early stages of inflammatory signaling; blocking TLR activation could potentially reduce the downstream production of potent adaptive immunity-associated molecules, leading to decreased inflammatory cell infiltration and inflammatory signaling in the sinonasal mucosa.

Glycosaminoglycans (GAGs) are a class of innate immune system modulators known to inhibit multiple inflammatory mediators and serve roles in chemokine signaling, mucosal surface repair, and mucociliary clearance in the airways.[20–23] GAGs, such as heparan and chondroitin sulfate, heparin, and hyaluronic acid (HA), are polysaccharides composed of unique, repeating disaccharide units. With the exception of HA, GAGs are found covalently attached to a protein core as proteoglycans that are expressed on epithelial and mucosal cells in the upper airway and in the extracellular matrix.[24,25] Heparin has shown anti-inflammatory and mucolytic properties in the treatment of respiratory diseases, such as allergic rhinitis, cystic fibrosis, asthma, and chronic pulmonary obstructive disorder; however, its potent anti-coagulant activity has limited its therapeutic potential in CRS.[22,26–29] Highly sulfated synthetic GAGs based on HA that do not possess anti-coagulant activity have shown to effectively coat and penetrate the sinonasal mucosa in mice to reduce inflammation in a model of acute rhinosinusitis and other inflammatory conditions.[30–33]

**Fig 1:**
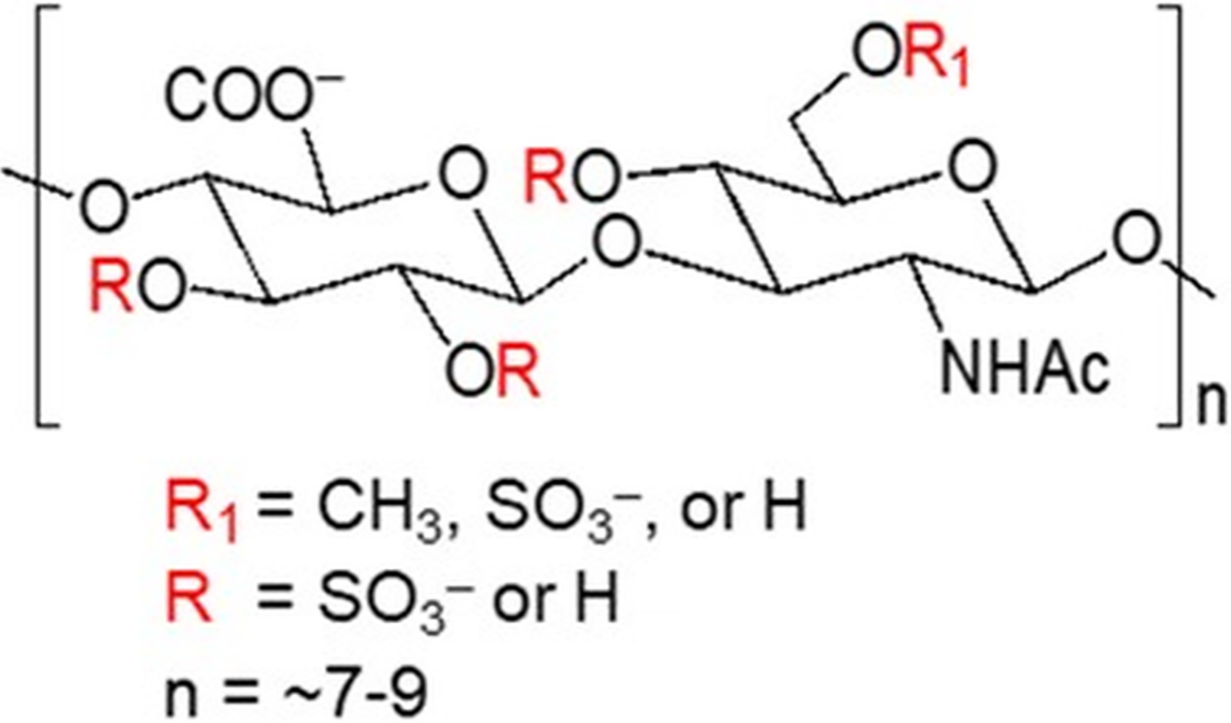
The chemical structure of synthetic glycosaminoglycan, GM-1111.

In the present study, we hypothesized that a synthetic GAG, GM-1111 (Fig 1), can effectively reduce sinonasal inflammation and the innate and adaptive inflammatory tissue biomarkers associated with a murine model of allergy-induced eosinophilic CRS.

## Materials and methods

### Study compounds

GM-1111 was synthesized as previously described by Ricerca Biosciences (Concord, OH),[34] *Aspergillus fumigatus* (*A*. *fumigatus*) extracts were obtained from Stallergenes-Greer Laboratories (Lenoir, NC), and Imject^TM^ Alum Adjuvant was obtained from ThermoFisher Scientific (Pittsburgh, PA).

### Animals and experimental design

Male BALB/c mice (8–10 weeks old) were purchased from Charles River Laboratories (Santa Clara, CA) and housed in a barrier system at the University of Utah’s Comparative Medicine Center. All animal procedures were performed according to the Guide for the Care and Use of Laboratory Animals [35] and approved by the Institutional Animal Care and Use Committee at the University of Utah (Protocol 15–11021).

**Fig 2:**
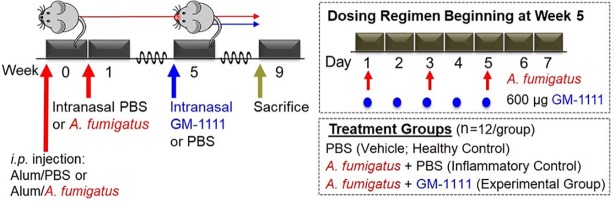
Study design to examine the anti-inflammatory properties of GM-1111 in a murine model of CRS. At week 0, control animals (PBS group) were sensitized with an intraperitoneal (*i*.*p*.) injection of PBS vehicle in alum adjuvant, whereas the *A*. *fumigatus* + PBS (red arrow) and *A*. *fumigatus* + GM-1111 (blue arrow) groups received *A*. *fumigatus* extracts in alum adjuvant. After 1 week, the animals were subjected to an 8-week regiment (3 times weekly) of intranasal PBS or *A*. *fumigatus* extracts. At week 5, intranasal treatment of GM-1111 or PBS was initiated (5 times weekly) and continued for 4 weeks with continued *A*. *fumigatus* extract administration (upper right inset). At week 9, whole blood was collected, and the animals were sacrificed and examined for histologic changes and inflammatory tissue biomarkers associated with CRS.

CRS was generated in BALB/c mice using repeated intranasal administration of *A*. *fumigatus* extracts, according to Lindsay *et al*.[36] Modifications to the original animal model, in addition to details regarding the treatment groups and dosing regimen used in this study, are illustrated in Fig 2. The animals were separated into the following treatment groups: PBS (vehicle; healthy control, n = 12), *A*. *fumigatus* + PBS (inflammatory control, n = 12), and *A*. *fumigatus* + GM-1111 (experimental group, n = 12). At week 0, the PBS group was sensitized with an intraperitoneal (*i*.*p*.) injection of 200 μL of a 1:1 mixture of PBS/Imject^TM^ Alum Adjuvant, whereas the *A*. *fumigatus* + PBS and *A*. *fumigatus* + GM-1111 groups received 200 μL of 20,000 PNU/mL *A*. *fumigatus* extracts/Imject^TM^ Alum Adjuvant. After 1 week, the animals were subjected to a 4-week course of 3 times per week intranasal administration of 10 μL of PBS (Sigma Aldrich, St. Louis, MO) or *A*. *fumigatus* extracts (20,000 PNU/mL PBS) per nostril while under light isoflurane anesthesia. At week 5, intranasal treatment of 10 μL GM-1111 (300 μg dose in PBS per nostril) or PBS was initiated (5 times per week) and continued for 4 weeks, during which the animals were awake. During weeks 5–9, animals received continued administration of *A*. *fumigatus* extracts 3 times per week to maintain a chronic inflammatory environment with acute exacerbations. On those days that required the administration of both *A*. *fumigatus* and GM-1111, the animals were treated at least 4 hours apart to control for any direct interactions between *A*. *fumigatus* and GM-1111 at the sinonasal epithelium. At week 9, whole blood was collected, and the animals were sacrificed and examined for histologic changes and inflammatory tissue biomarkers associated with CRS. Body weight measurements and behavioral (clinical) signs (1. nasal erythema, 2. nose scratching, 3. sneezing, and 4. apneic events or gasping) were recorded 3 times a week throughout the study. The data are reported as the number of observed clinical signs after week 4, when treatment with GM-1111 or PBS vehicle was initiated, for each animal per treatment group.

### Histology

All study animals were sacrificed at week 9 by exsanguination under deep isoflurane anesthesia, and the heads were trimmed and fixed in 4% neutral formalin (Ted Pella, Redding, CA) for 48 hours. Fixed tissues were decalcified using 14% ethylenediaminetetraacetic acid (EDTA, pH 7.2) (Sigma Aldrich, St. Louis, MO) for 2 weeks, followed by sectioning of sinonasal tissues under an Olympus FSX100 stereoscope (Olympus Inc., Center Valley, PA). The tissue sections were embedded in paraffin and coronal tissue sections (4 μm thick) were mounted on the slides. Tissues were stained with hematoxylin and eosin (H&E) or immunolabeled, as next described. Tissue embedding, paraffin sectioning, and H&E staining were performed by HistoTox Labs (Boulder, CO).

### Complete blood cell count (CBC)

Whole blood samples were collected in EDTA-coated microvettes (Sarstedt, Inc., Sparks, NV) by cardiac puncture prior to exsanguination. CBC and manual differential white blood cell counts were determined by SRI Biosciences (Menlo Park, CA).

### Immunohistochemistry

Sinonasal tissues were deparaffinized in xylene (3 x 10 min) and rehydrated using decreasing concentrations of ethanol (100%, 95%, and 70%, 2 x 5 min) and ddH_2_O (2 x 5 min). Unless stated, all staining reagents were obtained from and used as recommended by Vector Laboratories (Burlingame, CA). All sinus tissues were examined and imaged under an Olympus BX43 upright microscope (Olympus Inc., Pittsburgh, PA) using an EOS Rebel T2i digital SLR camera (Canon Inc., Melville, NY).

*Acid mucopolysaccharides (goblet cells) and dividing cells (tissue remodeling)*: Tissues were stained using a NovaUltra^TM^Alcian Blue/Nuclear Fast Red Solution Staining Kit (IHC World, Woodstock, MD) following the supplier’s instructions and then subjected to staining for proliferating cell nuclear antigen (PCNA). Heated antigen retrieval was performed in citrate buffer (pH 6.0), and tissues were blocked in BLOXALL and then subjected to IHC detection of mouse anti-mouse PCNA (1:6000; 2 h at room temperature) (Abcam, Cambridge, MA) using Mouse on Mouse (M.O.M.^TM^) and ImmPACT DAB Peroxidase Kits.

*TLR2*: Heated antigen retrieval was performed in Tris-OH buffer (pH 8.0), and tissues were blocked in BLOXALL, subjected to IHC detection of mouse anti-mouse TLR2 (1:250; 2 h at room temperature) (MyBioSource, San Diego, CA) using Mouse on Mouse (M.O.M.^TM^) and ImmPACT DAB Peroxidase Kits, and counterstained with hematoxylin.

*CD4+ cells (T cells)*: Heated antigen retrieval was performed in Tris-OH buffer (pH 8.0), and tissues were blocked in BLOXALL, subjected to IHC detection of rabbit anti-mouse CD4 (1:1000; overnight at 4°C) (Abcam, Cambridge, MA) using ImmPRESS^TM^ HRP anti-rat IgG and ImmPACT DAB Peroxidase Kits, and counterstained with hematoxylin. The severity of CD4+ cell infiltration was determined by counting and averaging the number of CD4+ cells present per six random high-power field images in a similar coronal section for each animal and assigning a severity index of 0 (no), 1 (focal), 2 (mild, n<10), 3 (moderate, 10<n<30), or 4 (severe, n>30) with respect to the number/presence of CD4+ cells.

*Eosinophils*: Heated antigen retrieval was performed in citrate buffer (pH 6), and tissues were blocked in BLOXALL and then subjected to IHC detection of rat anti-mouse major basic protein-1 monoclonal antibody (MBP; 1:200; overnight at 4°C) (Mayo Clinic, Scottsdale, AZ)[37,38] using ImmPRESS^TM^ HRP anti-rat IgG and ImmPACT DAB Peroxidase Kits. Eosinophil infiltration severity was determined by counting the number of MBP+ cells present in a similar coronal section of each animal.

### Quantification of serum IgE

Serum IgE was determined using an ELISA MAX^TM^ Deluxe Mouse IgE Kit (Biolegend, San Diego, CA) following the manufacturer’s instructions. IgE concentrations were standardized to the International System of Units of IgE per liter as recommended for clinical reporting and are represented as the mean ± standard deviation for each treatment group.[39]

### Gene expression profiling

Gene expression levels were determined using the paraffin embedded tissues. Sinonasal tissues were subjected to tissue punching (ethmoturbinate tissue), nucleic acid extraction, and gene expression analyses using Inflammation V2 gene panels (NanoString Technologies, Seattle, WA), which were performed by the Biorepository and Molecular Pathology Core and the Molecular Diagnostic Core at the Huntsman Cancer Institute (University of Utah, Salt Lake City, UT). The panels consisted of 248 inflammatory and 6 housekeeping genes. After RNA extraction, the samples were subjected to overnight hybridization at 65°C; 5 μL of RNA per sample were added to 8 μL of nCounter reporter probes and 2 μL of nCounter capture probes. The post-hybridization target genes were quantified using an nCounter Digital Analyzer and nSolver Software. Six housekeeping genes (*cltc*, *gapdh*, *gusb*, *hprt*, *pgk1*, and *tubb5*) were used for background subtraction and normalization of the raw mRNA transcript copy counts for gene and sample. After normalization, ratios of the difference in the means of the log-transformed normalized data to the square root of the sum of the variances of samples in the three treatment groups were generated. A two-tailed t-test on the log-transformed normalized data was then performed assuming unequal variance. The data are reported as the mean ± the standard error of the mean gene transcript copy in each treatment group.

### Statistical analysis

Statistical analyses were performed using Prism 6 for Windows (GraphPad Software; La Jolla, CA). For all datasets, statistical comparisons were made by one-way Analysis of Variance, followed by Tukey’s post hoc test to adjust for multiple comparisons with a *p* value of ≤ 0.05 being statistically significant.

## Results

### Observations of murine clinical signs

**Fig 3:**
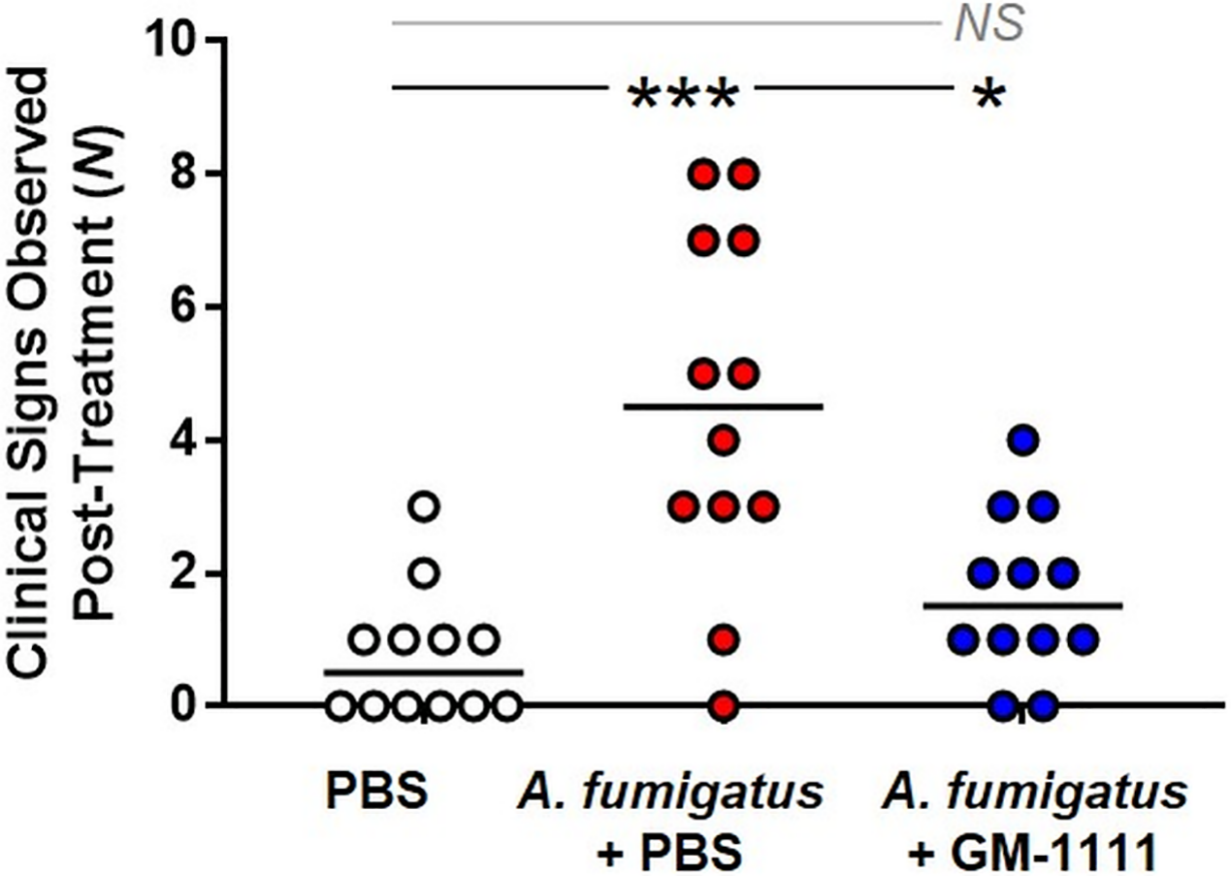
GM-1111 treatment significantly reduces clinical signs of CRS in mice. Clinical signs include nasal erythema and edema, itchy nose, sneezing, and difficulty breathing (n = 12 per group). The data are represented as the number of recorded clinical signs measured after beginning treatment with GM-1111, reported for each animal per treatment group with the median. *p<0.05; ***p<0.001; NS: Not Significant.

Symptoms commonly associated with CRS-associated inflammation include nasal discharge, congestion, nose irritation, and overall discomfort.[1,2,40] These clinical signs also develop in mice when sensitized to intranasal *A*. *fumigatus* extract administration. Sneezing, difficulty breathing, and erythema of the nose were monitored and recorded throughout the model and regarded as potential clinical signs associated with CRS. Fig 3 demonstrates the total number of clinical signs observed after initiating treatment with GM-1111 or PBS vehicle for each animal per treatment group. Compared to healthy animals, there was a significant increase in the number of recorded clinical signs in the vehicle-treated, *A*. *fumigatus* group (Fig 3; *p*<0.001). By contrast, the clinical signs in the GM-1111-treated animals were significantly reduced (*p*<0.05), suggesting that GM-1111 administration might provide nasal symptom relief.

### Inflammation-induced sinonasal tissue damage

**Fig 4:**
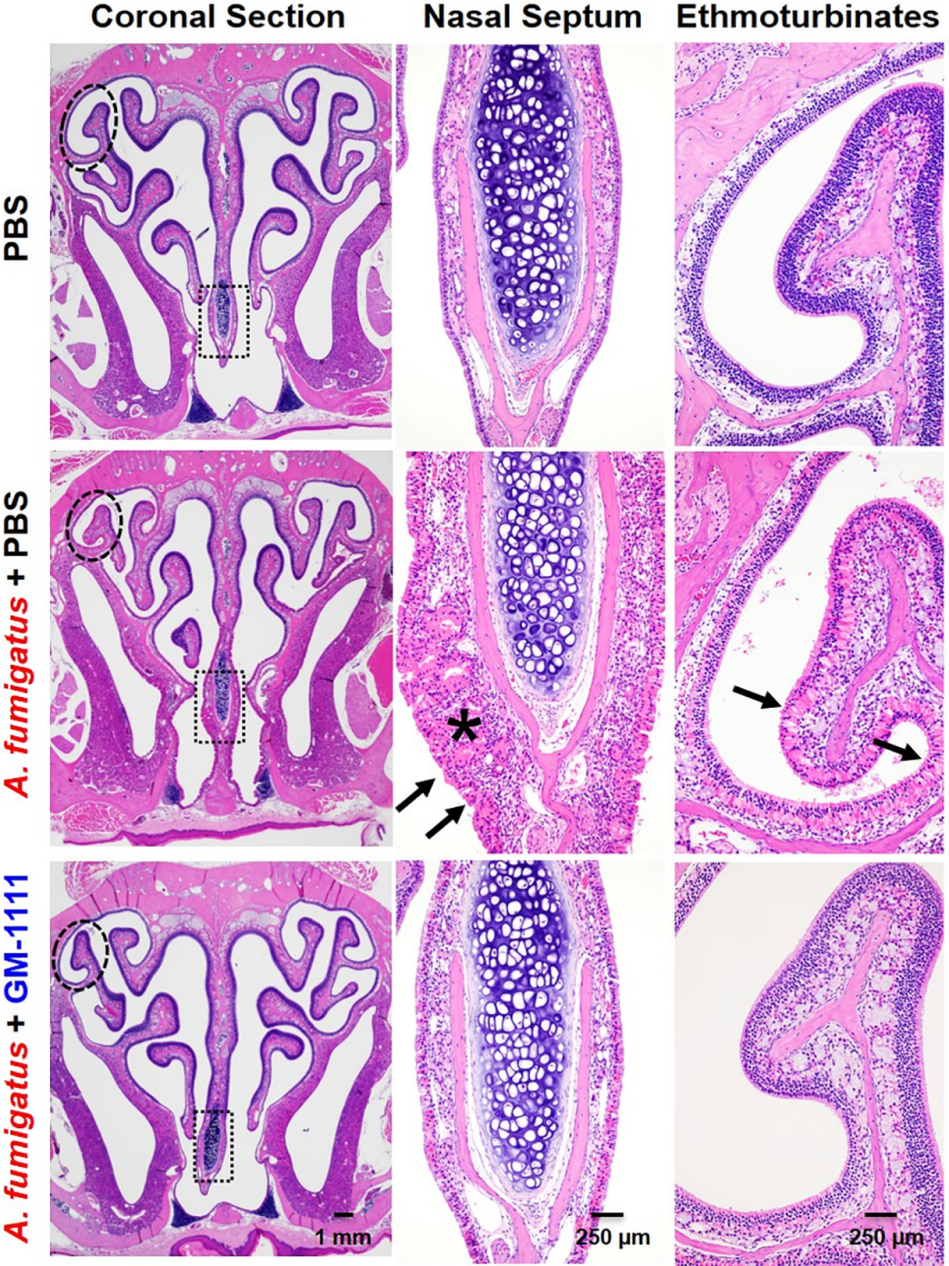
GM-1111 reduces CRS-associated sinonasal inflammation in mice. Microscopic images of sinonasal tissues stained with hematoxylin and eosin show coronal sections and respective higher magnification images of the indicated region of the nasal septum (box) and ethmoturbinate tissue (circle). *A*. *fumigatus* administration resulted in marked degenerative changes and loss of cilia in all epithelial layers (arrows), increased inflammatory cell infiltration, and thickening of the nasal septum (star). These changes were much less pronounced in animals treated with GM-1111. Histopathological interpretations were generated using images from all animals (n = 12 per treatment group) with representative images demonstrated.

**Fig 5:**
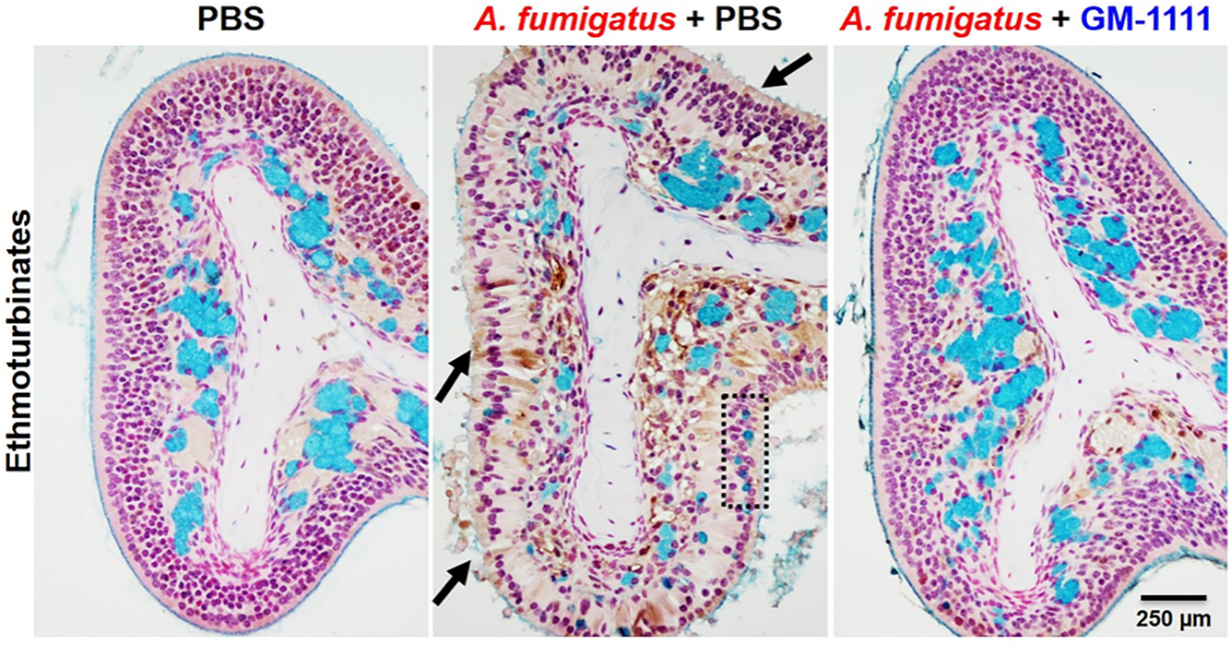
GM-1111 reduces CRS-associated changes in mucus-secreting cells in the sinuses of mice. The ethmoturbinates from *A*. *fumigatus*-treated mice stained with Alcian Blue (turquoise = acid mucopolysaccharides) show the loss of luminal surface mucus lining and ciliated cells (arrows) and decreased alcian blue staining, indicating a reduction in glandular contents with concomitant goblet cell hyperplasia in the epithelium (dotted box). Anti-PCNA immunolabeling (brown = dividing cells) demonstrates increased cellular activity to regenerate in tissues from *A*. *fumigatus*-treated mice. These changes were much less pronounced in animals treated with GM-1111. Histopathological interpretations were generated using images from all animals (n = 12 per treatment group) with representative images demonstrated.

CRS is clinically characterized by sinonasal inflammation with epithelial breakdown, mucosal thickening, goblet cell hyperplasia, and increased inflammatory cell infiltration.[8,13] Fig 4 demonstrates hematoxylin and eosin-stained tissue sections composed of respiratory (nasal septum) and specialized respiratory and olfactory (ethmoturbinates) epithelium and mucosa to highlight the global tissue damage with *A*. *fumigatus* administration and the effects of GM-1111 to reduce tissue damage. Compared to the sinonasal tissues from healthy controls, tissues from the vehicle-treated disease group were characterized by degenerative changes in all epithelial layers (arrows), loss of cilia, marked inflammatory cell infiltration, and thickening of the nasal septum (star). Similar changes were also observed in the ethmoturbinates with atrophic epithelial layer erosion, loss of cilia, and increased inflammatory cell infiltration. These tissue changes were accompanied by increased goblet cell hyperplasia (box) and lack of luminal surface mucus lining when evaluated with alcian blue staining for acid mucopolysaccharides (arrows) (Fig 5). Global increases in tissue remodeling were also evident from the elevated protein levels of proliferating cell nuclear antigen (PCNA, brown signal), which is expressed by dividing cells (Fig 5).[41] In contrast, the sinonasal tissues from animals treated with GM-1111 demonstrated reduced degenerative changes, including reduced septal thickening, inflammatory cell infiltration, and goblet cell hyperplasia. The increased appearance of ciliated epithelial cells was also observed in these tissues, with similar levels of tissue regeneration (PCNA signal) to those from healthy controls (Figs 4 and 5), suggesting that GM-1111 treatment may induce the restoration of proper mucociliary function and tissue homeostasis.

### Expression of TLR2 and TLR4, adaptor molecules, and downstream cytokines involved in innate immunity

**Fig 6:**
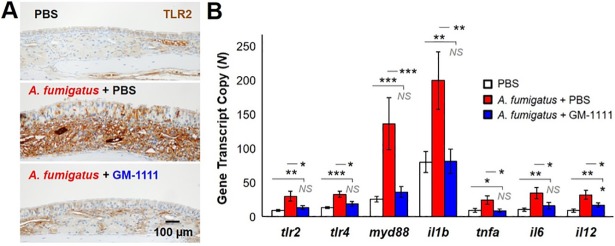
GM-1111 reduces TLR2 and TLR4 protein and NFκB-regulated gene expression of downstream inflammatory cytokines associated with innate immunity. The sinonasal tissues from *A*. *fumigatus*-treated animals demonstrate increased (**A**) protein (n = 10, PBS; n = 12, *A*. *fumigatus* + PBS; n = 12, *A*. *fumigatus* + GM-1111) and (**B**) gene expression of TLR2 and TLR4 compared to controls and animals treated with GM-1111. The gene expression of adaptor molecule *myd88* and cytokines *il1b*, *tnfa*, *il6*, and *il12* associated with TLR2 and TLR4 activation were also elevated in the sinus tissues of animals treated with *A*. *fumigatus* (n = 12, PBS; n = 8, *A*. *fumigatus* + PBS; n = 12, *A*. *fumigatus* + GM-1111). Treatment with GM-1111 showed significant reductions in these innate immune biomarkers compared to disease controls. The genes from each group were normalized to *cltc*, *gapdh*, *gusb*, *hprt*, *pgk1*, and *tubb5* and plotted as the mean ± SEM of gene transcript copy in each treatment group. The images demonstrating TLR2 are representative for each treatment group. *p<0.05; **p<0.01; *p*<0.001; NS: Not Significant.

Consistent with innate immune response activation by *A*. *fumigatus* extracts, elevated levels of TLR2 protein (Fig 6A) and TLR2 and TLR4 gene expression (Fig 6B; *p*<0.01 and *p*<0.001, respectively) were observed in the sinonasal tissues harvested from *A*. *fumigatus*-treated animals compared to those from healthy controls.[42] The gene expression of *myd88*, an adaptor molecule of TLRs required for nuclear factor kappa B (NF-κB) complex activation and subsequent production of inflammatory cytokines (*il1b*, *tnfa*, *il6*, and *il12*) downstream of TLR2 and TLR4 were also increased in the tissues of the disease group (*p*<0.001 to *p*<0.05). Expression of these biomarkers was significantly reduced, most of which was driven back to baseline, with GM-1111 treatment, suggesting that GM-1111 had an inhibitory effect on TLR2 and TLR4 activation and their regulation of cytokine production.

### Inflammatory cell counts and expression of IgE and cytokines involved in allergy and Th2-mediated adaptive immunity

**Fig 7:**
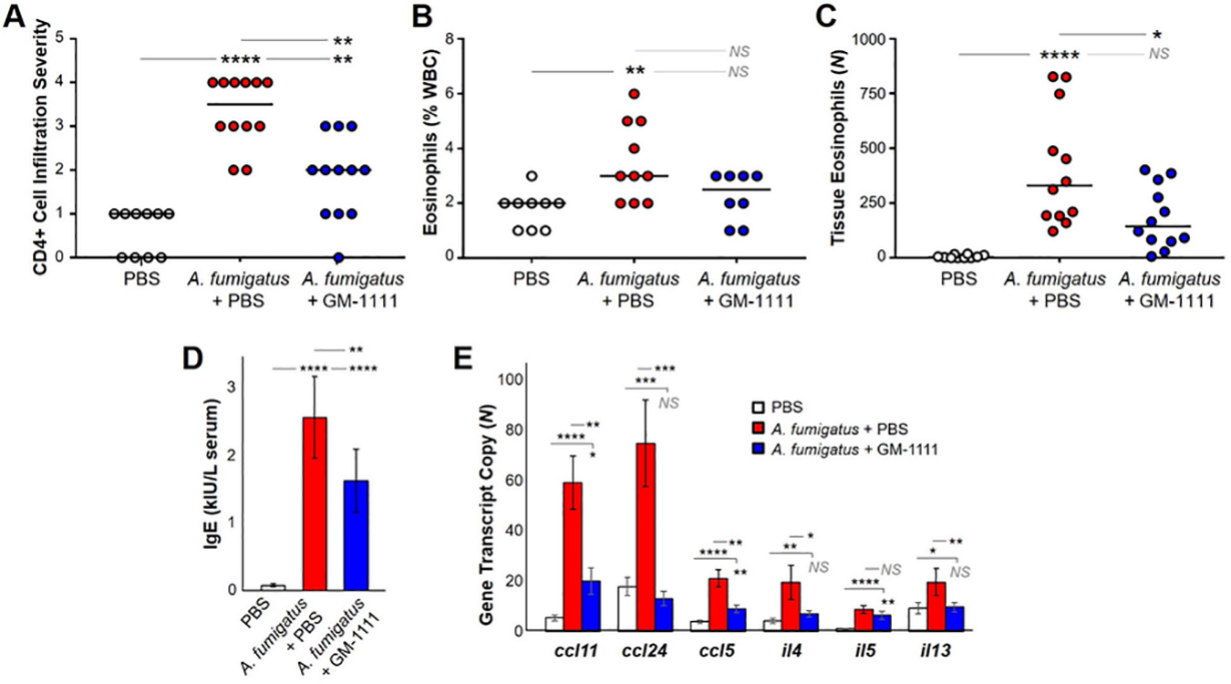
GM-1111 reduces blood and tissue biomarkers associated with allergy and Th2-mediated adaptive immunity. Compared to controls, animals treated with *A*. *fumigatus* exhibited significant increases in (**A**) CD4+ T cells found in the sinonasal tissues (*p*<0.0001) (n = 10, PBS; n = 12, *A*. *fumigatus* + PBS; n = 12, *A*. *fumigatus* + GM-1111), expressed as an infiltration severity index based on numbers of CD4+ cells counted per high power field, (**B**) blood eosinophils (% WBC) determined by CBC count (*p*<0.01) (n = 9, PBS; n = 10, *A*. *fumigatus* + PBS; n = 8, *A*. *fumigatus* + GM-1111), (**C**) eosinophils found in the sinonasal tissues (*p*<0.0001) (n = 10, PBS; n = 12, *A*. *fumigatus* + PBS; n = 12, *A*. *fumigatus* + GM-1111), (**D**) serum IgE levels (kIU/mL) (n = 10, PBS; n = 5, *A*. *fumigatus* + PBS; n = 10, *A*. *fumigatus* + GM-1111), and (**E**) eosinophil-associated cytokine and chemokine gene expression (*ccl11*, *ccl24*, *ccl5*, *il4*, *il5*, and *il13*) (n = 12, PBS; n = 8, *A*. *fumigatus* + PBS; n = 12, *A*. *fumigatus* + GM-1111). Treatment with GM-1111 showed significant reductions in the influx of CD4+ T cells (*p*<0.01) and eosinophils (*p*<0.05), as well as IgE levels (*p*<0.0001) and gene expression levels (*p*<0.01 or *p*<0.001) compared to disease controls. The genes from each group were normalized to *cltc*, *gapdh*, *gusb*, *hprt*, *pgk1*, and *tubb5* and plotted as the mean ± SEM of gene transcript copy in each treatment group. *p<0.05; **p<0.01; ***p<0.001; ****p<0.0001; NS: Not Significant.

To examine the endotypes commonly associated with *A*. *fumigatus* induction, we quantified the abundance of eosinophils in the blood, as well as Th2 cells and eosinophils residing in the sinonasal tissues by performing complete blood count analysis (% of total white blood cells (WBCs)) and IHC detection of CD4 and eosinophil-specific granule protein, MBP, respectively. Compared to healthy controls, there was a significant increase in the presence of CD4+ cells, expressed as the cell infiltration severity index based on the number of CD4+ cells counted and averaged from six random high-power field images per animal, in the sinonasal tissues collected from the *A*. *fumigatus* group (Fig 7A; *p*<0.0001). Similarly, significant increases in eosinophil numbers were observed in the blood (Fig 7B; p<0.01) and sinonasal tissues (Fig 7C; p<0.0001) harvested from the disease group when compared to controls. In contrast, GM-1111-treated animals demonstrated a reduction in blood eosinophils (Fig 7B; NS) with significant decreases in tissue-residing CD4+ cells (Fig 7A; *p*<0.01) and eosinophils (Fig 7C; *p*<0.05), suggesting that GM-1111 treatment inhibits CD4+ and eosinophil infiltration into the sinuses.

Consistent with an allergic etiology, significant increases in serum IgE protein levels were measured in mice treated with *A*. *fumigatus* extracts *vs*. controls (Fig 7D; *p*<0.0001).[43–45] A significant 36% reduction in IgE was measured in animals treated with GM-1111 (*p*<0.01). We additionally quantified the gene expression of eosinophil-specific cytokines and chemokines in sinonasal tissues of each animal. *il5* is essential for eosinophil survival, whereas *il4* and *il13* activate eosinophils in a positive feedback loop and are also secreted by activated epithelial cells. Eotaxins (*ccl11* and *ccl24*) and regulated on activation normal T cell expressed and secreted (RANTES or *ccl5)* are potent chemoattractants that mediate eosinophil chemotaxis and allergy signaling. Significant increases in *ccl11*, *ccl24*, *ccl5*, *il4*, *il5*, and *il13* gene transcript numbers were demonstrated in the sinonasal tissues of *A*. *fumigatus*-administered animals when compared to healthy controls (Fig 7E; *p*<0.0001 to 0.05). Apart from *il5*, expression of these gene transcripts was significantly reduced with GM-1111 treatment (*p*<0.001 to 0.05), suggesting that GM-1111 is effective in reducing key inflammatory cells and cytokines involved in allergy and Th2-mediated adaptive immune signaling.

## Discussion

GM-1111 is a promising solution to existing and emerging therapies for treating CRS. Taking advantage of the inherent inflammation-modulating effects of GAGs, GM-1111 is synthetically tailored to retain desirable anti-inflammatory and mucolytic properties while selectively engineering out the less desirable ones, such as anti-coagulant activity and being easily degraded.[46,47] The backbone of the GM-1111 is shared with HA, which serves important roles in mucociliary clearance and repairing mucosal surfaces,[20,21] whereas the hydroxyl groups of the disaccharide units are chemically sulfated to obtain the anti-inflammatory and mucolytic effects of heparin.[26] Due to its anionic structure, GM-1111 is highly water soluble and can be readily formulated in physiological buffers for increased sinonasal epithelial and mucosal penetration,[48] a key advantage over intranasal corticosteroids, which demonstrate less than 3% distribution and penetration within the sinuses.[49] The global reductions in local and systemic inflammatory biomarkers, as well as marked improvements in histologic tissue changes suggest that GM-1111 effectively absorbs to the sinonasal epithelium and penetrates the mucosa through topical administration.

**Fig 8:**
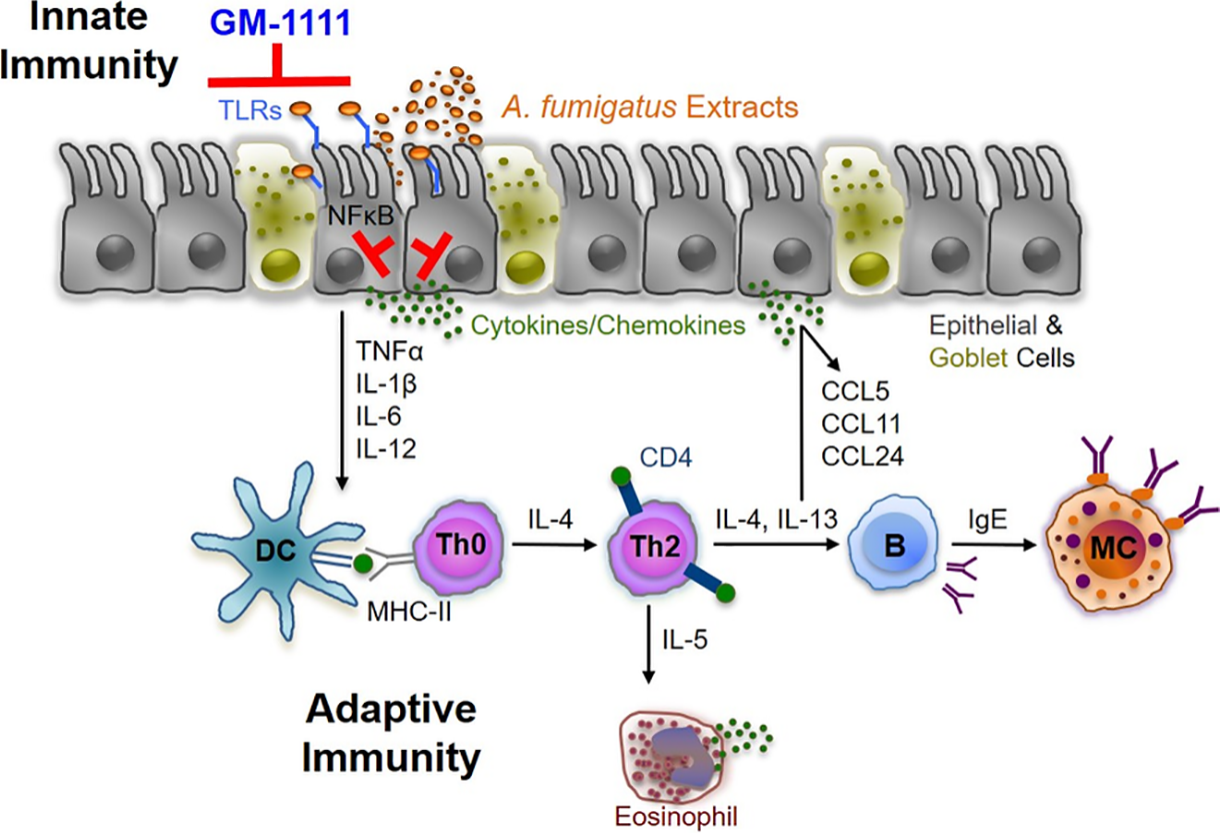
Schematic illustrating the proposed mechanism by which GM-1111 reduces allergic CRS-associated inflammation. GM-1111 blocks the innate immune response through TLR activation, thereby suppressing the expression of proinflammatory cytokines, chemokines, and allergy signaling molecules and inhibiting the chemotaxis of CD4+ T cells and eosinophils, which amplify adaptive immune signaling.

Our data demonstrate that GM-1111 markedly reduces the expression of TLR2 and TLR4 and inflammatory cell migration and invasion into the sinonasal mucosa and epithelium, resulting in the local reduction of cytokine gene expression. We have previously shown that synthetic GAGs strongly block TLR2-induced activation of NFκB and the resulting transcription of proinflammatory mediators in macrophages and human embryonic kidney cells.[32] This outcome occurs most likely through the inhibition of TLR and its agonists, pathogen-associated molecular patterns (PAMPs). As particularly relevant targets for inflammatory respiratory diseases, the TLRs are a family of membrane-bound pattern recognition receptors localized on sinonasal and immune cells that respond to endogenous and exogenous stimuli to maintain homeostasis and serve critical roles during early inflammatory signaling to initiate the innate immune response.[15,16,19] Recent investigations have demonstrated that TLR2 and TLR4 are upregulated in patients with CRS and that inappropriate regulation of signaling contributes to the increased inflammation observed in CRS.[18] TLR2 and TLR4 activation result in the downstream production and release of numerous pro-inflammatory cytokines and chemokines that in turn attract inflammatory cells, leading to increased inflammatory cell infiltration through the adaptive immune response. Based on the gene expression data herein, GM-1111 suppresses innate immune response-associated cytokine expression, possibly through its inhibition of TLR-mediated NFκB activation and translocation to the nucleus to regulate the transcription of such pro-inflammatory mediators. By blocking early inflammatory signaling and initiation of the innate immune response through TLR2 and TLR4, GM-1111 is indirectly reducing the initiation of the adaptive immune response, which may lead to decreased inflammatory cell infiltration, epithelial cell barrier degeneration, and mucociliary dysfunction (Fig 8). Further *in vitro* studies are warranted to directly address this hypothesis in correlation to the animal study performed herein.

Although their function is not well understood, numerous studies have implicated eosinophils as key modulators of Th2-driven inflammation in noninvasive forms of allergy-dominated endotypes and in patients with CRSwNP. Eosinophilic inflammation has been associated with recalcitrant inflammatory disease due to the difficulty in controlling the patients’ underlying inflammation.[8,50,51] We therefore examined and quantified the gene expression of eosinophil-specific cytokines and chemokines in sinonasal tissues. IL-4 and 13 are important immunoregulatory cytokines involved in T-cell differentiation into a Th2 phenotype with induction of IgE synthesis and activation of eosinophils in an active positive feedback loop. The resulting activation of T lymphocytes induces further synthesis and secretion of IL-4 and IL-13, which are involved in the chemotaxis of eosinophils and mast cells. Given that eosinophils are primary IL-5Rα expressing cells, IL-5 synthesis and release is critical in modulating eosinophilic inflammation from maturation to survival.[52] Further enhancement of eosinophilic inflammation is regulated by CCR3, a high affinity cell surface receptor to eotaxins (CCL11 and CCL24) and RANTES (CCL5), all of which are potent chemoattractants mediating eosinophil chemotaxis. Herein, we demonstrated that topical treatment with GM-1111 significantly reduced eosinophil counts both in the blood and in sinonasal tissues. Furthermore, we observed a significant reduction in eosinophil-specific cytokine and chemokine gene expression in sinonasal tissues relative to the *A*. *fumigatus* group, with marked decreases in potent chemoattractant *ccl11* and *ccl24* transcripts. This is consistent with emerging therapeutic development efforts that have focused on targeting eosinophils to reduce inflammation and polyp size through the generation of injectable monoclonal antibodies against IL-5, IL-5Rα, and IL-4/IL-13.[53–55] The ability to inhibit eosinophil infiltration, activation, and resultant pro-inflammatory signaling locally, as well as systemically, has promising implications for GM-1111 as a new effective, topical treatment for patients with eosinophilic CRS. Future studies will be directed at determining whether GM-1111 has an inhibitory effect on eosinophil and mast cell degranulation or on reducing polyp size using *ex vivo* nasal polyp tissues and cells isolated from patients with CRSwNP.

A limitation to these *in vivo* studies lies within the choice of animal and model. As CRS is a heterogeneous disease with multiple etiologies, several models and model organisms exist to study it, however, with no defined consensus. Other model and model organisms for CRS that cause an inflammatory response include inoculation of bacteria and induction of ovalbumin with *S*. *aureus* enterotoxin B.[56,57] To date, the A. *fumigatus* mouse model for CRS is one of the most validated models available to develop reproducible inflammation and examine the pathophysiology and treatment options for recalcitrant forms of eosinophilic CRS. We recognize that there are differences in mouse sinus anatomy and inflammatory profiles to humans and these differences should be considered when examining the results.[58,59]
